# Migration and Health

**DOI:** 10.1155/2012/621914

**Published:** 2012-12-22

**Authors:** Katarina Hjelm, Björn Albin, Rosa Benato, Panayota Sourtzi

**Affiliations:** ^1^School of Health and Caring Sciences, Linnaeus University, Sweden; ^2^School of Health Sciences, City University London, UK; ^3^Department of Public Health, Faculty of Nursing, University of Athens, Greece

Global migration is extensive and ongoing and is today an international process and an international issue affecting every country in the world [1]. As a result of global migration many countries have been transformed into multicultural societies with an increased chance of encountering migrants or those with a migrant history in health care. This can be a challenge for health professionals as disease patterns, health-related beliefs and behaviours, ability to express symptoms and signs of health and illness as well as expectations on health care providers and nursing care may differ significantly. An understanding and knowledge of the relationship between migration and health is limited; however, it is urgently needed all over the world. 

International migration is increasing and it is estimated that today 190 states in the world are points of origin, transit, or destination for migrants. It is also estimated that the number of migrants has risen from 82 million in 1970 to 175 million in 2000, more than doubling over the course of thirty years [2], and further into 214 million in 2010 [1]. The reasons for the increase of migration are many; in some instances these are linked to better opportunities for work and better life standards, in others to safeguarding one's life from turbulent political situations or environmental disasters. For example, one important reason for the increase of migration in Europe has been disintegration of the Soviet Union [1]. 

Health can be influenced by migration and several earlier ecological studies have examined health in relation to lifestyle factors and certain diagnoses of cancer in different migrant groups [3]. The increase in international migration also makes it important to study the consequences on different elements and levels of the host countries' society using a variety of research designs.

The studies in this edition reflect perspectives from different countries such as Sweden, Canada, the UK, and the United States, countries to which migration is high. 

Two of the studies are longitudinal epidemiological studies focusing on the situation in Sweden for migrants in a long-term perspective concerning mortality and the utilization of health care (Albin et al.). The other four studies investigate the health situation for particularly vulnerable groups among migrants, women and migrant farmworkers (Babatunde et al., MacDonnell et al., Guruge et al., and Bail et al.). The latter uses a qualitative approach with focus groups interviews, grounded theory, narrative interviews in a case-study, and structured interviews.

Women's mental health is highlighted in three of the studies; in one it is related to postnatal depression, in another it is discussed in relation to a history of violence and the third one is in relation to health promotion and empowerment. The fourth qualitative study illustrates how isolation from family and community, as well as perceived invisibility within institutions, for example, in health care and social service, affect health and well-being of migrant farm workers. 

Although the variety of migrant populations and study designs is limited, we hope that this compilation provides readers and researchers with an overview of contemporary and relevant research activity and helps them find new information in the area of migration and health. We also hope we can inspire others to use different migrant groups and research methods appropriate to the research question and further broaden the existing knowledge base so that health care professionals have the possibility to adapt their care to the needs of migrant populations.

*Katarina Hjelm* *Katarina Hjelm* *Björn Albin* *Björn Albin* *Rosa Benato* *Rosa Benato* *Panayota Sourtzi* *Panayota Sourtzi*

## References

[1] International Organisation for Migration (2010). *World Migration Report 2010: The Future for Migration: Building Capacities for Change*.

[2] International Organisation for Migration (2005). *World Migration 2005: Cost and Benefit of International Migration*.

[3] Willett W (1998). *Nutritional Epidemiology*.

